# 3D printed osteochondral scaffolds: design strategies, present applications and future perspectives

**DOI:** 10.3389/fbioe.2024.1339916

**Published:** 2024-02-15

**Authors:** Ge Liu, Xiaowei Wei, Yun Zhai, Jingrun Zhang, Junlei Li, Zhenhua Zhao, Tianmin Guan, Deiwei Zhao

**Affiliations:** ^1^ School of Mechanical Engineering, Dalian Jiaotong University, Dalian, China; ^2^ Department of Orthopedics, Affiliated Zhongshan Hospital of Dalian University, Dalian, China

**Keywords:** 3D printing, osteochondral, tissue engineering, biomaterials, biomimetic scaffolds

## Abstract

Articular osteochondral (OC) defects are a global clinical problem characterized by loss of full-thickness articular cartilage with underlying calcified cartilage through to the subchondral bone. While current surgical treatments can relieve pain, none of them can completely repair all components of the OC unit and restore its original function. With the rapid development of three-dimensional (3D) printing technology, admirable progress has been made in bone and cartilage reconstruction, providing new strategies for restoring joint function. 3D printing has the advantages of fast speed, high precision, and personalized customization to meet the requirements of irregular geometry, differentiated composition, and multi-layered boundary layer structures of joint OC scaffolds. This review captures the original published researches on the application of 3D printing technology to the repair of entire OC units and provides a comprehensive summary of the recent advances in 3D printed OC scaffolds. We first introduce the gradient structure and biological properties of articular OC tissue. The considerations for the development of 3D printed OC scaffolds are emphatically summarized, including material types, fabrication techniques, structural design and seed cells. Especially from the perspective of material composition and structural design, the classification, characteristics and latest research progress of discrete gradient scaffolds (biphasic, triphasic and multiphasic scaffolds) and continuous gradient scaffolds (gradient material and/or structure, and gradient interface) are summarized. Finally, we also describe the important progress and application prospect of 3D printing technology in OC interface regeneration. 3D printing technology for OC reconstruction should simulate the gradient structure of subchondral bone and cartilage. Therefore, we must not only strengthen the basic research on OC structure, but also continue to explore the role of 3D printing technology in OC tissue engineering. This will enable better structural and functional bionics of OC scaffolds, ultimately improving the repair of OC defects.

## Graphical Abstract

**Figure:**
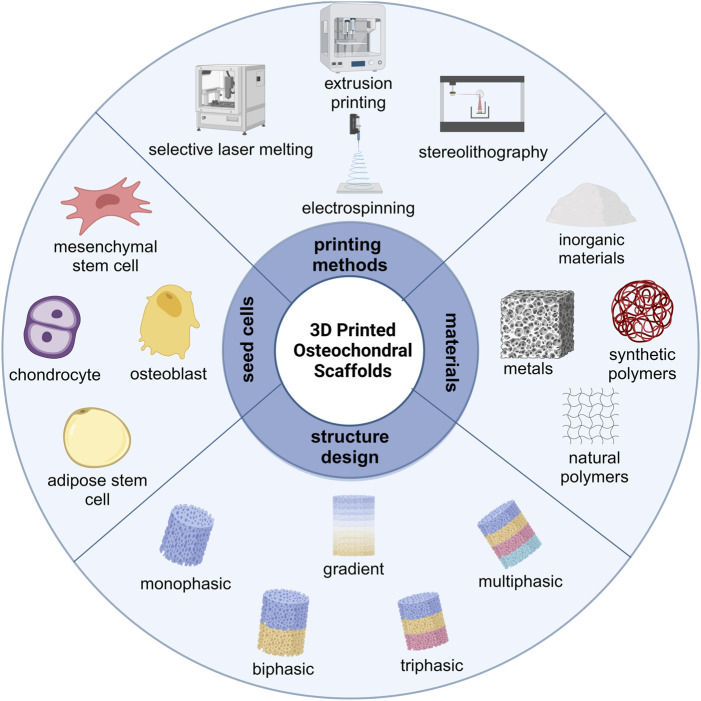
Schematic illustration of the key elements in 3D printed OC scaffolds. Figure created with BioRender.com.

## 1 Introduction

The OC unit is a highly organized tissue that includes superficially layered articular cartilage and underlying subchondral bone connected by calcified cartilage interface region (Goldring and Goldring, 2016). OC performs the essential functions of transmitting and distributing mechanical loads to the skeletal system during movements (Sophia Fox et al., 2009). OC defects may result from acute traumatic injury, such as sports-related trauma or falls, or from diseases, such as osteochondritis dissecans. They most commonly occur in the knee and ankle joints, but are also found in other sites such as the hands and spine (Zhou et al., 2020). When injured OC is untreated or inadequately treated, the joint may irreversibly deteriorate, leading to chronic pain and impaired mobility, thereby seriously affecting the quality of life of the individual, and causing a serious socioeconomic burden on society (Hannon et al., 2014; Dee et al., 2022).

Because OC defects involve cartilage, OC interface, and subchondral bone, and the tissue structure and composition of each part are different, the treatment of OC damage is still a global clinical problem (Ye et al., 2014). The current clinical treatment for OC defects is mostly surgical treatments, including debridement, microfracture, bone marrow stimulation, autologous/allogeneic OC transplantation, autologous chondrocyte transplantation, and so on (Bowland et al., 2015; Yasui et al., 2017; Salzmann et al., 2018). Although these traditional treatment strategies have their corresponding advantages, their inherent disadvantages are also evident. For example, the OC tissues repaired by debridement, microfracture, and bone marrow stimulation are all fibrocartilage, which is far from articular cartilage in nature (Clouet et al., 2009). Compared with hyaline cartilage, fibrocartilage has poorer mechanical properties and biological properties, and gradually degenerates over time, resulting in permanent loss of structure and function, and severe pain, which seriously affects the quality of daily life of patients (Aigner and Stöve, 2003). The donor area of autologous cartilage transplantation is less, and there are problems such as immune rejection and disease transmission in allogeneic cartilage transplantation (Assenmacher et al., 2016; Gobbi et al., 2020). Therefore, there is a lack of practical and effective treatment methods for OC defects clinically.

In recent years, the development of tissue engineering technology offers a novel approach to the treatment of OC defects. Tissue engineering technology aims to combine seed cells and growth factors with material scaffolds to repair the structure and function of damaged tissues (Sherman et al., 2017). Cartilage and bone tissue engineering have been researched since 1990, and most developed approaches are based on highly simplistic, unitary representations of each tissue type. With the advent of additive manufacturing technology, 3D printing has developed rapidly, providing a new construction strategy for the preparation of engineered living tissue, which can realize the gradient biomimetic of OC tissue (Abdollahiyan et al., 2020; Lafuente-Merchan et al., 2022). 3D printing strategies enhance control over the microstructural environment of engineered tissues by modulating materials, structural design, and distribution of biological components. This capability is particularly relevant to generalizing tissue interfaces, including the OC unit, as each corresponding tissue can be tailored to achieve a specific architectural framework and bioactivity (Groll et al., 2016; Viola et al., 2021).

With the rapid development of 3D printing technology, many researchers are committed to using printing technology to develop multilayer OC scaffolds with biomimetic structures. Therefore, this review focuses on the original published researches on the application of 3D printing technology to the repair of entire OC units, especially the latest articles on 3D printed OC scaffolds. We first introduce the gradient structure and biological properties of articular OC tissue, highlighting the gradient characteristics of OC tissues from cell types, tissue components and mechanical properties. Then we summarized the considerations for developing 3D printed OC scaffolds, including material types, fabrication techniques, structural design and seed cells. The introduction to biomaterials is more extensive, detailing the advantages and limitations of natural and synthetic polymers suitable for the cartilage layer and inorganic and metal-based materials suitable for the bone layer. The various 3D printing technologies that have been developed (including based on powders, fibers, liquids and light sources) are classified and summarized, and the scaffolds construction strategies and current development levels of various 3D printing technologies are compared. We analyzed the structural design of 3D printed OC scaffold in detail. From the perspective of material composition and structural design, the classification, characteristics and latest research progress of discrete gradient scaffolds (biphasic, triphasic and multiphasic scaffolds) and continuous gradient scaffolds (gradient material and/or structure, and gradient interface) are summarized. The types and advantages of seed cells commonly used in OC scaffolds are mainly introduced, with special emphasis on the different ways of loading seed cells and the repair effects of 3D printed OC scaffolds. Finally, we evaluated the future application prospect and development direction of this field. The schematic illustration of the key elements in 3D printed OC scaffolds is summarized in Graphical Abstract.

## 2 Structure of OC tissue

OC tissue is composed of articular cartilage, calcified layer, and subchondral bone. It is the key structure to maintain the normal activities of human joints. The stability of its function is the prerequisite for the normal function of joints (Boyde, 2021; Wei and Dai, 2021). OC tissue has specific gradient structures and biological properties. Therefore, to design an OC biomimetic gradient scaffold, it is necessary to understand the composition, structure, and function of the OC unit.

### 2.1 Articular cartilage

Articular cartilage is mainly hyaline cartilage that covers the surface of movable joints. The average thickness of human articular cartilage depends on the location within the joint and the age of the patient. Articular cartilage varies in thickness from 3 mm to 7 mm at different anatomical sites (Hunziker et al., 2002; Bhosale and Richardson, 2008; Antons et al., 2018). Articular cartilage has a glass-like appearance, smooth surface, and excellent elasticity. It has the characteristics of wear resistance, anti-friction, and lubricating joints, and also plays the role of impact resistance and vibration cushioning (Thambyah et al., 2006). Articular cartilage has no blood vessels, nerves, and lymphatic vessels. Its nutrition mainly comes from synovial fluid and subchondral bone blood vessels. It cannot self-regenerate and is only composed of water, chondrocytes, and extracellular matrix (ECM) (Jiang and Tuan, 2015; Armiento et al., 2018).

**FIGURE 1:**
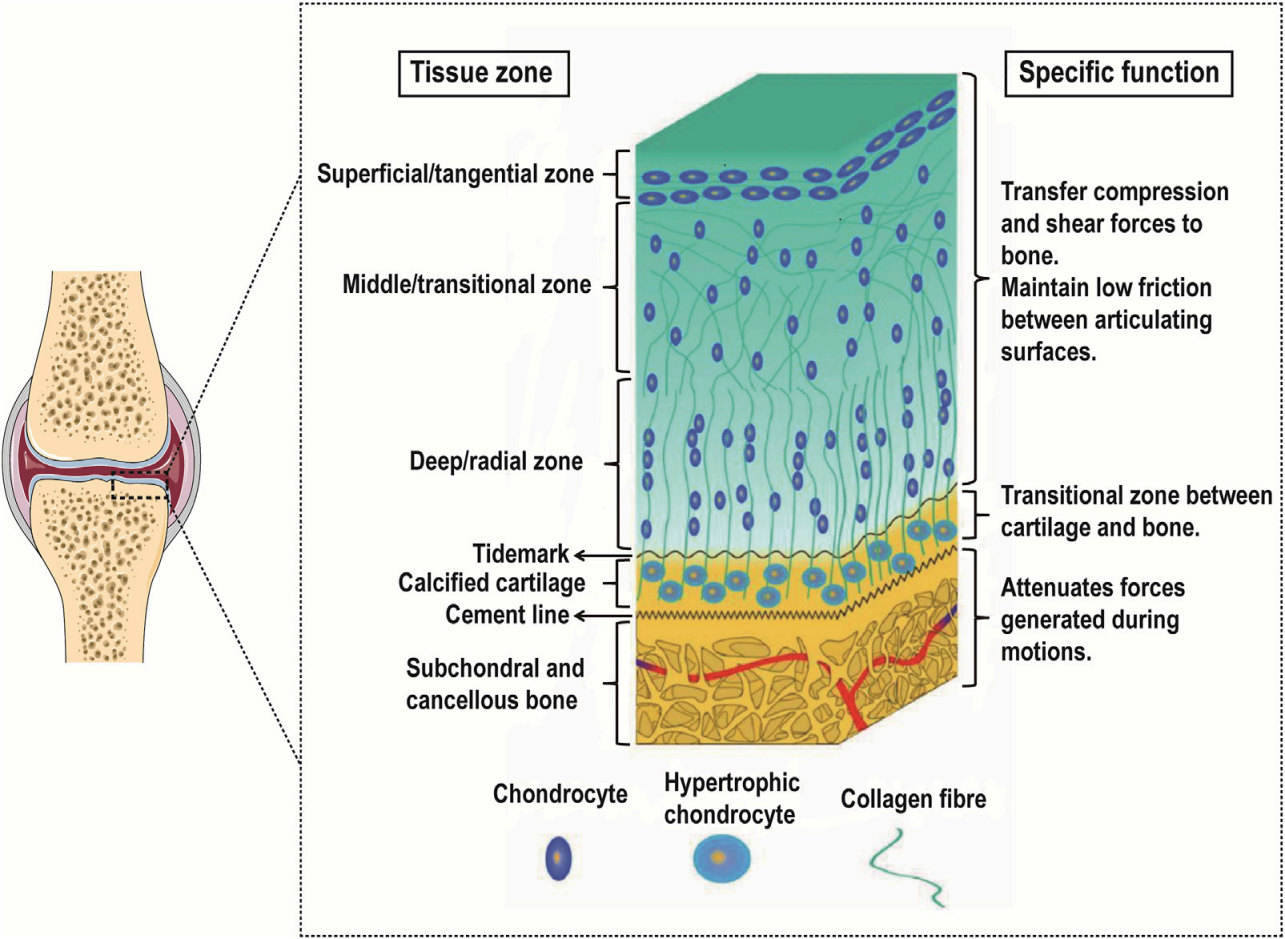
Gradient schematic illustrating the different zones of OC tissue and their specific functions. Reproduced with permission from (Khanarian et al., 2014). While the OC tissue is made up of articular cartilage (including the superficial zone, the middle zone, the deep zone, and the calcified cartilage) and subchondral bone, each of these tissues is heterogeneous. Especially in the articular cartilage, each zone within varies in cell size, number, and orientation as well as collagen fiber size, type, and orientation.

Articular cartilage can be divided into microstructures from top to bottom: superficial layer, middle layer, deep layer, and calcified layer. There is a tidal line structure at the junction of the deep layer and the calcified layer. Its main function is to connect the relatively soft cartilage layer with the relatively hard calcified layer. Below the calcified layer is the subchondral bone platform, and the two layers are interlaced anchoring. The structure formed at the junction is also called a cement line (Lemoine et al., 2020) (Figure 1).

The superficial layer accounts for 10%–20% of the hyaline cartilage layer. The distribution density of chondrocytes in this layer is the highest, most of them are flat, and the cell density is relatively high; the direction of collagen fibers is parallel to the surface of cartilage and the diameter of collagen fibers is relatively thin (4–12 nm), while the arrangement is relatively dense. The middle layer accounts for 40%–60% of the hyaline cartilage layer. The chondrocytes in this layer are round, the cell density is reduced, the diameter of collagen fibers is thickened (9–60 nm) and the direction of arrangement is random. The deep layer accounts for 30%–40% of the hyaline cartilage layer. The chondrocytes in this layer are elongated and some are nearly spherical. They are arranged in columns. Compared with the superficial layer and the middle layer, the cell density is lower, and the diameter of collagen fibers is thick (60–140 nm) and arranged perpendicular to the surface of articular cartilage (Martin et al., 2007; Becerra et al., 2010; Di Luca et al., 2015; Kwon et al., 2016; Panadero et al., 2016).

As the transition zone of OC tissue, the density of chondrocytes in the calcified cartilage further decreases and shows hypertrophy and tissue calcification appears at the same time. These hypertrophic chondrocytes mainly synthesize type X collagen. This area also contains type I/II collagen, proteoglycans, and carbonated hydroxyapatite. These collagen fibers are anchored to the subchondral bone and serve to fix the cartilage and subchondral bone (Fan et al., 2021; Xu et al., 2022).

On the whole, from top to bottom in the cartilage tissue, the cell density and type II collagen show a decreasing trend, and the type X collagen and type I collagen gradually appear and show an increasing trend, while the degree of calcification gradually increases. Therefore, the mechanical properties of cartilage also show regional depth-dependent changes, and its compressive modulus gradually increases from the superficial layer to the deep layer, that is, from 0.2 MPa to 6.44 MPa (Gao et al., 2014; Kabir et al., 2021), as shown in Table 1.

**TABLE 1:** Biochemical gradients of osteochondral tissue.

Qsteochondral tissue		Ratio (%)	Cell phenotypes	Collagen composition, diameter and direction	Mechanical property
Cartilage	Superficial zone	10–20	Flattened chondrocytes	II (+++)	The compressive modulus of cartilage: from the superficial zone to the deep zone increasing from 0.2 MPa to 6.44 MPa
			Highest density	4–12 nm in diameter, parallel to articular surface	
	Middle zone	40–60	Slightly round chondrocytes	II (++)	
			Higher density	9–60 nm in diameter, randomly arranged	
	Deep zone	30–40	Columnar chondrocytes	II (+)、X (++)	
			Lower density	60–140 nm in diameter, perpendicular to the articular surface	
	Calcified zone		Hypertrophic chondrocytes	I (++)、II (+)、X (+++)	
			Lowest density	Fixed to subchondral bone	
Subchondral bone			Osteoblasts, osteoclasts, osteocytes, and MSCs	I (+++)	The compressive modulus of subchondral bone: value for cortical bone is 18–22 GPa, value for trabecular bone is 0.1–0.9 GPa
				Hydroxyapatite crystals are deposited on it	

“+”quantity represents the intensity change of collagen content, (+++) indicates the highest content, (+) indicates the least content.

### 2.2 Subchondral bone

The subchondral bone is located below the calcified layer and is a highly vascularized biomineralized connective tissue that bears the stress load from the cartilage, buffers mechanical loads on the joint shock, and maintains the normal shape of the joint (Stewart and Kawcak, 2018; Hu et al., 2021). The subchondral bone is composed of the subchondral bone platform composed of cortical bone and the subchondral cancellous bone composed of cancellous bone. Among them, cortical bone is composed of repeating bone units with low porosity and less vascularity. Cancellous bone consists of an interconnected framework of trabecular bone with irregular shapes and random orientations, forming a marrow-filled space with high porosity and rich blood vessels and nerves (Netzer et al., 2018).

Subchondral bone is composed of water (10%), organic components (30%) and inorganic components (60%). Water fills pores, and binds collagen fibers and mineral crystals. Organic ingredients include 90% type I collagen and 5% non-collagen, which play a very important role in the construction of bone matrix networks, cell signal transduction, and mineralization while providing flexibility and elasticity to bone tissue. The inorganic component is mainly hydroxyapatite crystals, which are formed by the precipitation of calcium phosphate minerals. They are deposited on type I collagen fibers and contribute to the rigidity and load-bearing strength of bone tissue (Kalia et al., 2014). Cells in bone tissue include osteoblasts, osteoclasts, osteocytes, and mesenchymal stem cells (MSCs). Osteoblasts are cells that form new bone and are also responsible for the synthesis of hydroxyapatite. Osteoclasts are involved in bone resorption. Osteocytes are the most common cell type in bone and regulate the interaction between osteoblasts and osteoclasts. MSCs can differentiate into bone cells and chondrocytes (Feng and McDonald, 2011). The mechanical properties of subchondral bone depend on the specific structure of bone tissue and have anisotropy. For example, the compressive modulus values of cortical bone and trabecular bone are 18–22 GPa and 0.1–0.9 GPa, respectively (Zhang et al., 2020b), as shown in Table 1. OC tissue shows a gradient transition from soft cartilage to hard subchondral bone. Therefore, in OC tissue engineering, it is of great significance to realize this gradient transition.

## 3 3D printed OC repair materials

Currently, researchers have developed a variety of materials for 3D printing to prepare OC engineering scaffolds. According to the composition of the materials, there are four main categories: natural polymers, synthetic polymers, inorganic materials, metals, and composite materials in which the above four categories are mixed (Nooeaid et al., 2012; García-Gareta et al., 2015), as shown in Table 2.

**TABLE 2:** Common osteochondral scaffold materials used for 3D printing.

material type	Osteochondral scaffold materials for 3D printing
Natural polymers	Polysaccharides (PS): alginate (ALG), chitosan (CS), hyaluronic acid (HA), agarose (AG), cellulose (CE), gellan gum (GG), etc.
	Proteins: collagen (COL), gelatin (GEL), fibronectin (FN), silk fibroin (SF), etc.
Synthetic polymers	Polyethylene glycol (PEG), polycaprolactone (PCL), polylactic acid (PLA), polyglycolic acid (PGA), poly (lactic-co-ethanolic acid) (PLGA), etc.
Inorganic materials	Calcium-phosphorus-based bioceramics: β-tricalcium phosphate (β-TCP), hydroxyapatite (HA), etc.
	Calcium-silica-based bioceramics: calcium silicate (CS), bioactive glass (BAG), etc.
Metals	Titanium (Ti), tantalum (Ta), magnesium (Mg), etc.
Composite materials	The combination of the above materials

### 3.1 Natural polymers

Natural polymers are usually used in the form of hydrogels and include polysaccharides and proteins. Among them, polysaccharides include alginate (Chen P. et al., 2018), chitosan (Shoueir et al., 2021), hyaluronic acid (Yontar et al., 2019), agarose (Armstrong et al., 2022), cellulose (Cordeiro et al., 2023), gellan gum (Choi et al., 2020), etc. and proteins include collagen (Marques et al., 2019), gelatin (Echave et al., 2019), fibronectin (Nulty et al., 2021), and silk fibroin (Ni et al., 2020), etc. These natural polymer networks are capable of holding large amounts of water, thus creating a fully hydrated 3D environment comparable to the natural ECM (Ahmed, 2015; Sánchez-Téllez et al., 2017). This environment can support the adhesion, proliferation, and differentiation of various cells (Zhu and Marchant, 2011). However, natural polymers usually have weak mechanical properties. Although they can fulfill the mechanical property requirements for cartilage repair, their mechanical strength is insufficient for bone repair, which can lead to deformation of the load-bearing region (Fuchs et al., 2020).

Most natural polymers used for 3D printing often improve their mechanical properties through crosslinking (Lin et al., 2021). Common crosslinking strategies include physical crosslinking (Liao et al., 2017), chemical crosslinking (Xiao et al., 2019), light crosslinking (Lee M. et al., 2020), UV crosslinking (Hong et al., 2020), energy electron irradiation crosslinking (Tang et al., 2021), and enzymatic crosslinking (Wu et al., 2022). Alternatively, these natural polymers can be modified in a way to improve mechanical properties. They are modified into photosensitive hydrogel materials, e.g., gelatin to gelatin methacryloyl (GelMA), hyaluronic acid to hyaluronic acid methacrylate (HAMA), which are categorized as semi-synthetic materials and widely used for OC repair (Pepelanova et al., 2018; Dienes et al., 2021). In addition, strategies to overcome mechanical limitations include compositing with other classes of materials (e.g., synthetic polymers or bioceramics, etc.) to improve their mechanical properties and enhance their bioactivity (Gao et al., 2019; Diloksumpan et al., 2020).

### 3.2 Synthetic polymers

Synthetic polymers are polymeric materials prepared based on chemical synthesis, which have better mechanical properties and adjustable biodegradability due to their controllable sequences (Lu et al., 2001). They are widely used in OC tissue engineering due to their better processability and plasticity, which make them more suitable for 3D printing. Among them, polyethylene glycol (PEG) (Zhu S. et al., 2020), polycaprolactone (PCL) (Du et al., 2017) polylactic acid (PLA) (Yao et al., 2017), polyglycolic acid (PGA) (Niederauer et al., 2000), and poly (lactic-co-ethanolic acid) (PLGA) (Liang et al., 2018) are commonly used as raw materials for constructing OC tissue engineering scaffolds.

With the advantages of low melting temperature, good processability, biodegradable mechanical properties, and relatively low cost, PCL has been approved by the FDA and is widely used in the field of tissue engineering (Li and Tan, 2014; Xu et al., 2021; Dethe et al., 2022). PEG is the most widely used synthetic polymer approved by the FDA, which can be implanted as a scaffold in the body due to its unique physicochemical properties, biodegradability, and non-immunogenicity (Zhang J. et al., 2016; Yang J. et al., 2021). PLA and PLGA are also used in all stages of OC scaffolds. For example, Critchley et al. compared the mechanical properties of the ratio of PCL, PLA, and PLGA in cartilage phase scaffolds (Critchley et al., 2020). However, synthetic polymers also have significant limitations; their hydrophobic surfaces are not conducive to cell adhesion and proliferation; most of the degradation products are acidic, hindering cell differentiation, predisposing to inflammatory reactions at the implantation site, and lacking OC-inducing properties (Jurak et al., 2021). Therefore, future research strategies should focus on establishing an effective combination of natural and synthetic bioinks to capitalize on the advantages of both materials while offering the possibility of OC regeneration solutions.

### 3.3 Inorganic materials

Inorganic materials mainly refer to bioceramics, including calcium-phosphorus-based bioceramics and calcium-silica-based bioceramics. Among them, calcium-phosphorus-based bioceramics mainly refer to β-tricalcium phosphate and hydroxyapatite, etc., and calcium-silica-based bioceramics mainly refer to calcium silicate and bioactive glass, etc. (Albulescu et al., 2019; van Rijt et al., 2022). They have good bioactivity and osteoinductive properties and are mainly used in the bone layer and to a lesser extent in the calcified cartilage layer (Zafar et al., 2019). These materials naturally exist as brittle powders, thus limiting their ability to form independent porous structures on their own (Yousefi et al., 2014). Each material, formulation, source, and synthesis method in this category have different levels of osseointegration, biomineralization, osteoinduction, and osteoconductivity (Baino et al., 2015; Deng et al., 2019). In addition, the surface of such scaffolds can absorb osteoinductive factors and/or ions and continuously release them to modulate the surrounding environment and promote the differentiation of MSCs, thereby promoting bone formation *in vivo* (Ma et al., 2018).

Hydroxyapatite, β-tricalcium phosphate, and bioactive glass are the most commonly used bioceramics (Ribas et al., 2019). These materials have good biocompatibility, high osteoconductivity, and osteoinductivity, and can promote the production of bone-like apatite *in vivo*. Therefore, they are better integrated with the surrounding bone tissues and increase the bonding strength of the material to the bone tissue (Ielo et al., 2022). However, these materials have low fracture toughness, high brittleness, and high difficulty in material preparation. They are often used in combination with natural/synthetic polymers. Such composite materials can be prepared into porous scaffolds for cell attachment and proliferation by using 3D printing technology, which can be applied to bone repair in non-load-bearing and load-bearing areas (Castilho et al., 2014).

### 3.4 Metals

Metals that can be used for 3D printing include titanium, tantalum, magnesium, and their alloys, etc. They have good mechanical properties similar to the mechanical strength of bone and are mostly used in the repair of subchondral bone in the field of OC repair (Jing et al., 2020; Dutta and Roy, 2023; Jiao et al., 2023). However, the elastic modulus of solid metal materials is high, and the stress shielding effect will occur when implanted in the body, resulting in loosening or fracture of the implant. With the development of 3D printing technology, the research on porous metal scaffolds has gradually deepened. Compared with the traditional preparation process, 3D printing technology can realize personalized customized porous metal implants according to different anatomical morphologies, whose appearance is highly matched to the defect area, and the pore size, morphology, and porosity can be precisely controlled. Meanwhile, the elastic modulus of the implant is effectively reduced, to achieve better osseointegration (Wang X. et al., 2016).

At present, 3D printed porous titanium and its alloy-related standardized orthopedic implants have been approved for marketing in several products with precise clinical applications (Li et al., 2020; Losic, 2021). Tantalum is considered an ideal orthopedic endoprosthetic material for its excellent mechanical properties, corrosion resistance, and osteogenic properties. Scholars have already prepared porous tantalum prostheses by 3D printing technology and initially applied them to clinical applications with good follow-up results (Wang et al., 2020; Wang et al., 2021). Magnesium-based biodegradable alloy has excellent bioactivity and osteogenic ability. The porous magnesium scaffolds prepared by inkjet 3D printing have mechanical properties similar to cancellous bone and have obvious effects of promoting new bone regeneration in animals (Kraus et al., 2012; Liu et al., 2014). Therefore, magnesium-based biodegradable alloys are promising biomaterials for segmental bone defect repair. Although metallic materials have good mechanical properties, excessive mechanical properties are detrimental to cartilage repair and usually need to be applied in combination with other materials for OC tissue engineering.

Different classes of materials are used in combination with each other to form composite materials. 3D printing technology can be used to prepare porous scaffolds of composite materials. Due to the use of a variety of materials, the 3D printed scaffold has developed from single to multi-phase, which can better simulate the gradient structure of OC (Abdulghani and Morouço, 2019). Therefore, composite materials that combine the advantages of different materials will be the key to developing effective 3D printing strategies for regenerative OC interfaces.

## 4 Types of 3D printing technology

The preparation of OC scaffolds not only depends on excellent biomaterials, but also requires suitable manufacturing methods. Through the development of recent years, 3D printing has become a new technology for the fabrication of OC tissue engineering scaffolds (Daly et al., 2017; Wang S. et al., 2022). In 3D printing, the computer-aided design model guides the layer-by-layer manufacture of OC scaffolds, precisely controls the macroscopic shape and microscopic pore structure of the scaffolds, and meets the needs of individual customization. Therefore, more and more researchers are devoted to the research of 3D printing OC tissue engineering scaffolds (Brachet et al., 2023). Currently, a variety of 3D printing technologies have been developed, which can be broadly divided into powder-based (selective laser sintering and selective laser melting), fiber filament-based (fused deposition modeling, melt electro-writing and electrospinning), liquid-based (inkjet printing and extrusion printing), and light-source-based (stereolithography and digital light processing) 3D printing technologies (Bittner et al., 2018).

### 4.1 Powder-based 3D printing technologies

Selective laser sintering (SLS) and selective laser melting (SLM) both belong to the powder bed melting technology. The process of them is almost identical. They are both using the powder pre-positioned on the work platform as raw materials. The computer controls the two-dimensional scanning trajectory of the laser beam based on model slices, selectively molding the solid powder to form a dimension of the scaffold. This cycle is repeated, and the layers are stacked on top of each other to create the final three-dimensional scaffolds. The difference is that SLS can only partially melt the powders, whereas SLM can completely melt the powders (Gittard and Narayan, 2010; Wubneh et al., 2018).

Materials suitable for the SLS process can be polymers, bioceramics, or metal powders. Among them, bioceramics and metal powders are more widely used. Bioceramic powders are subjected to the SLS process with the addition of a binder, while metal powders can be sintered directly by the SLS process. The main drawbacks of SLS are low scaffold densities and large surface roughness, which need to be followed by hot isostatic pressing to improve the densities (Kamboj et al., 2021; Lupone et al., 2021). SLM is mainly used for metal powder printing, which allows precision molding, good surface quality, and good control of the aperture size of the molded parts. The surface quality is good and the pore size of the scaffolds can be well controlled (Yuan et al., 2019). SLM can be used to develop scaffolds with high porosity and various shapes, which do not require post-processing such as heat treatment (Trevisan et al., 2018).

### 4.2 Fiber filament-based 3D printing technologies

The printing processes of fused deposition modeling (FDM), melt electro-writing (MEW), and electrospinning (ES) all involve layer-by-layer deposition of fibrous filaments by a print nozzle (Doyle et al., 2021; Anandhapadman et al., 2022). FDM involves the formation of fibrous filaments for printing by heating and melting temperature-sensitive polymers and by extrusion (Gregory et al., 2023). MEW and ES control fibrous filaments by voltage and deposit them continuously onto the printing platform (Gonçalves et al., 2021; Loewner et al., 2022). The three printing techniques produce fiber filaments ranging in size from the micron level to the nanometer level. FDM typically produces hundred-micron-sized fibers, MEW typically produces micron-sized fibers, and ES typically produces nanometer-sized fibers (Brown et al., 2011; Wang S. J. et al., 2016; Han et al., 2021). Influenced by the printing principle and fiber filament thickness, FDW produces stiffer scaffolds that can be used for the simultaneous repair of OC phase (Hsieh et al., 2017; Hsieh et al., 2018; Zheng et al., 2019; Gong et al., 2020; Wang Y. et al., 2022), whereas MEW and ES usually produce softer scaffolds and are therefore mainly used for the repair of cartilage phase or calcified cartilage phase (Han et al., 2020; Kim et al., 2021; Steele et al., 2022).

The choice of materials must follow the printing principles of all three techniques. Commonly used materials are synthetic polymers such as PCL, PEG, PVA, PLGA, etc. However, inorganic-based materials such as HA are often added for bone repair, whereas natural polymers such as hyaluronic acid, collagen, and chitosan are added for cartilage repair (Ho et al., 2010; Hejazi et al., 2021; Kade and Dalton, 2021). Among them, PCL is often used as the base material in OC scaffolds, serving to mold the porous structure of the scaffold. In addition, FDM and MEW are solvent-free technologies, so the materials available are more limited. Whereas, ES is a solvent-based technology, so the materials used have increased, but the solvents used are usually toxic, and if there are residual toxins, the biocompatibility of the scaffolds may be compromised (Agarwal and Greiner, 2011; Wortmann et al., 2019).

### 4.3 Liquid-based 3D printing technologies

Inkjet printing (IP) and extrusion printing (EP) both use liquid materials with a certain viscosity to print scaffolds, but the printing principles of IP and EP are different. IP is a molding method in which bioink is extruded to form droplets by heat or piezoelectricity, and then continuously ejected from the nozzle to the print interface (Mandrycky et al., 2016). However, EP is a molding method in which bioink is continuously extruded from the material cylinder to the print platform in a pre-determined design by pneumatic pressure or mechanically (Ozbolat and Hospodiuk, 2016). IP and EP are relatively gentle molding methods that allow the printing of cell-loaded bioinks, thus enabling the precise positioning and distribution of multiple materials, cells, and bioactive factors, and laying the foundation for complex tissue and organ reconstruction (Murphy and Atala, 2014; Liu et al., 2017a; Loebel et al., 2017; Lee S. C. et al., 2020; Cui et al., 2020; Askari et al., 2021; Lawlor et al., 2021; Zandi et al., 2021).

Bioinks with suitable rheological behavior are available for both IP and EP. The main advantages of IP are low cost, fast printing speed, and high cell viability. However, the viscosity range of bioinks suitable for IP is very limited, high viscosity bioinks cannot be applied, and the cell density cannot be very high, or droplet formation will not be possible (Ozbolat and Hospodiuk, 2016). Therefore, the prepared scaffolds have insufficient mechanical strength, limited resolution, and rough surfaces, which cannot meet the mechanical strength required for bone repair and are usually used for cartilage repair (Liu et al., 2017b; Kyle et al., 2017). EP allows the use of a wider range of materials, including thermal polymers, hydrogels, bioceramics, etc. Different classes of materials or composites require the fine-tuning of printing parameters, such as temperature, extrusion pressure, printing speed, and degree of crosslinking or gelation, etc (Lee et al., 2013; Daly and Kelly, 2019; Bedell et al., 2022). Meanwhile, EP can prepare porous scaffolds at the micrometer level to allow cell proliferation and inward tissue growth. Smaller pores (<0.1–0.3 mm) contribute to the formation of new cartilage, while larger pores (>0.3 mm) promote bone tissue growth and are more suitable for simultaneous OC repair (Karageorgiou and Kaplan, 2005; Gupte et al., 2018; Li et al., 2021; Flégeau et al., 2022).

### 4.4 Light-based 3D printing technologies

Both stereolithography (SLA) and digital light processing (DLP) build 3D scaffolds by depositing materials layer by layer in a light-assisted manner. Its printing process is to place the liquid material and the build plate in the resin bath, adopt the light source tracking programming mode, and only crosslink the relevant design layer by layer until the scaffold is completed. The difference between SLA and DLP is the light source used. SLA uses a laser light source, while DLP uses a light source from a projector (Stansbury and Idacavage, 2016).

Both printing technologies are compatible with many materials. However, it is usually necessary to modify the material, which drastically changes the properties of the material and can limit its functionality (Li F. et al., 2018; Fiedor and Ortyl, 2020; Kumar et al., 2021). Materials suitable for use in OC scaffolds include PEG, GelMA, and TCP, which require mixing with any combination of photoinitiators, photoabsorbents, solvents, and/or dispersants to print (Castro et al., 2015; Aisenbrey et al., 2018; Chen P. et al., 2019; Zhou et al., 2019; Zhu S. et al., 2020; Schoonraad et al., 2021; Bedell et al., 2022). Both SLA and DLP can print porous structures at the tens of micrometer level, with the resolution between MEW/ES and EP (Wang Z. et al., 2015; Derakhshanfar et al., 2018). In addition, based on the printing principle that the whole layer of resin can be immediately polymerized, SLA and DLP have faster printing speeds and high-precision porous interconnected structures.

## 5 Classification of OC scaffolds

The main function of the scaffold is to provide an attached structural environment for tissue regeneration, cell loading, and release of bioactive factors. The emergence of 3D printing technology has made the structural design of the scaffold more complex, and can better simulate the gradient structural changes of OC tissue. According to the overall structure and performance of OC scaffolds, they can be roughly divided into five categories: monophasic scaffolds, discrete gradient scaffolds (biphasic scaffolds, triphasic scaffolds, and multiphasic scaffolds), and continuous gradient scaffolds. The “phase” represents its material composition ratio and structural design, etc. The 3D printed continuous gradient scaffolds is an emerging scaffold type, which will be highlighted in Chapter 6. The remaining four types of scaffolds are described in detail in this chapter.

### 5.1 Monophasic scaffolds

In recent years, the application of OC tissue engineering has achieved rapid development. The monophasic scaffold is one of the earliest applied repair techniques. Monophasic scaffolds refer to the entire scaffold whose material composition and structural design are the same. The scaffold material can be a single material component or a composite material component. Due to the uniformity of material composition and overall structure, monophasic scaffolds usually do not meet the needs of OC-integrated repair and are often used for bone repair or cartilage repair alone. For example, 3D printing-based metal porous materials have been widely used in the repair of bone tissue due to their compressive strength and elastic modulus similar to bone tissue. 3D printing-based porous metal scaffolds have been widely used in the repair of bone tissue due to their compressive strength and elastic modulus similar to bone tissue. For example, porous tantalum (Ta) scaffold is a novel implant material widely used in orthopedics including joint surgery, spinal surgery, bone tumor surgery, and trauma surgery. Guo et al., 2019 used SLM technology to manufacture a porous Ta scaffold, and the pore size was controlled to 400 μm. Compared with porous Ti6Al4V scaffolds, Ta scaffolds increased bone ingrowth and osseointegration. Hydrogel materials are similar to cartilage ECM and are often made into porous scaffolds by 3D printing for cartilage repair. For example, Sang et al. presented a photo-cross-linked ECM bioink composed of modified proteins and polysaccharides, including gelatin methacrylate, hyaluronic acid methacrylate, and chondroitin sulfate methacrylate. The results indicated that the photo-cross-linked ECM hydrogels possessed a suitable degradation rate and excellent mechanical properties, and the 3D bioprinted ECM scaffolds obtained favorable shape fidelity and effectively promoted cartilage regeneration (Sang et al., 2023). Bioceramics are often combined with natural or synthetic polymers to prepare 3D printable bioinks, which are more widely used in OC tissue engineering. Pei et al., 2017 introduced a two-step method of combining 3D printing and microwave sintering to fabricate a two-level hierarchical porous hydroxyapatite scaffold. This scaffold showed significant osteoinductive properties in animal experiments.

Monophasic scaffolds are not able to repair both bone and cartilage defects due to their homogeneous composition, but this limitation has been significantly improved with the development of 3D printing technology. When monophasic scaffolds are printed, cells or growth factors that are beneficial to osteogenesis and chondrogenesis are added to them to achieve simultaneous repair of bone and cartilage defects (Chen L. et al., 2019). Natalja et al. used a 3D fiber deposition technique to fabricate cell-laden, heterogeneous hydrogel constructs for potential use as osteochondral grafts. They encapsulated and printed human chondrocytes and osteogenic progenitors in alginate hydrogel yielding scaffolds of 1 2 cm with different parts for both cell types. Moreover, distinctive tissue formation was observed, both *in vitro* and *in vivo* at different locations within one construct (Fedorovich et al., 2012). There are also monophasic OC scaffolds, such as Zn/Co-MOF-functionalized β-TCP scaffolds, whose surfaces are functionally modified to enhance cartilage formation or osteogenic potential (Shu et al., 2023). Although monophasic scaffolds can satisfy the need for simultaneous repair of OC defects by adding cells or factors, their homogeneous composition prevents them from meeting the different mechanical properties required by OC tissue, which to some extent reduces their efficacy in the treatment of articular OC defects.

### 5.2 Biphasic scaffolds

**FIGURE 2:**
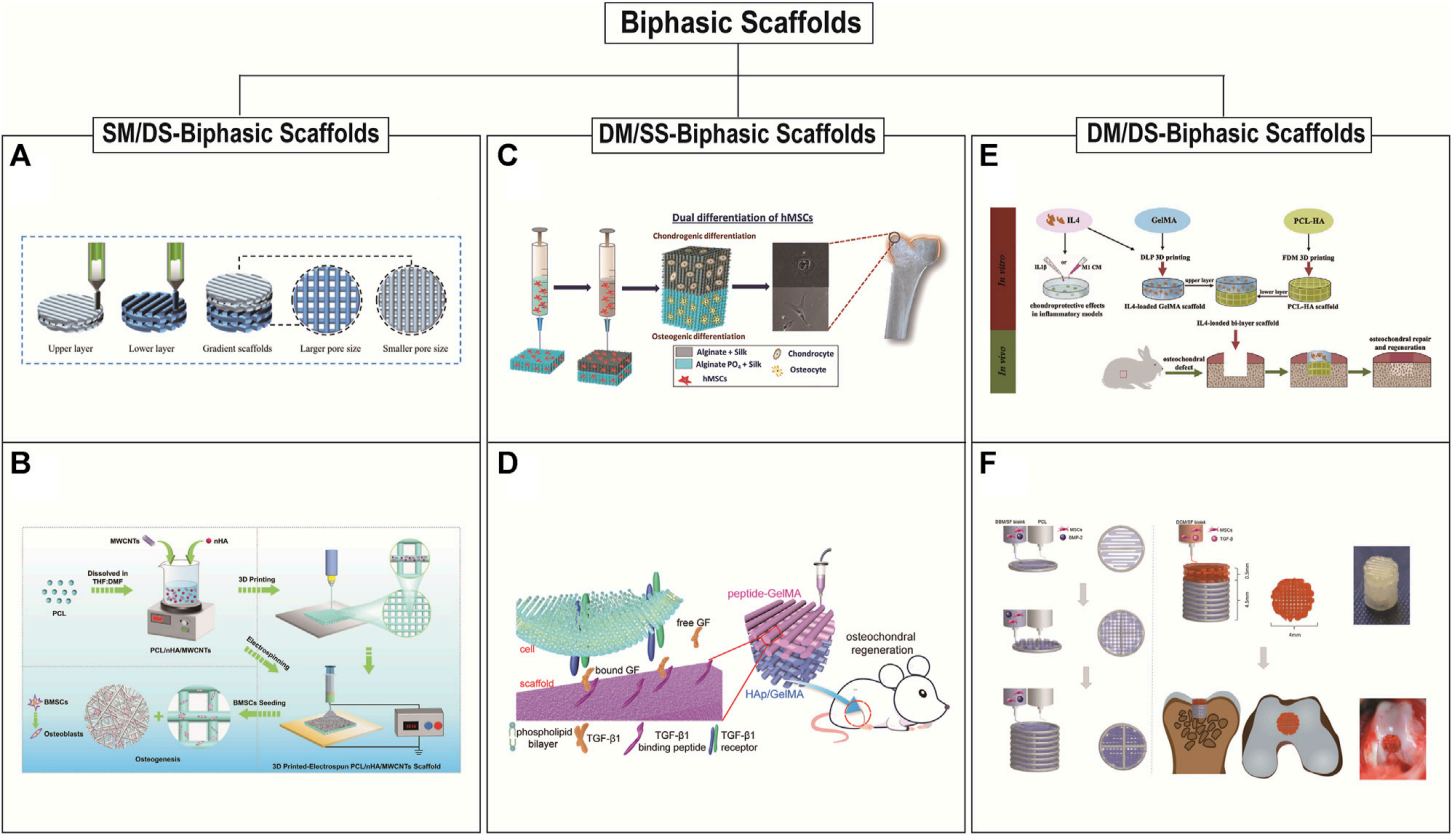
3D printed biphasic scaffolds for OC tissue engineering. Biphasic scaffolds include SM/DS (same material composition ratio but different structural design)-biphasic scaffolds **(A** and **B)**, DM/SS (different material composition ratio but same structural design)-biphasic scaffolds **(C** and **D)**, and DM/DS (different material composition ratio and different structural design)-biphasic scaffolds **(E** and **F)**. **(A)** 3D printing process of biphasic PCL scaffold with smaller size above and larger size below. Reproduced with permission from (Wang Y. et al., 2022). **(B)** Schematic illustration of the preparation of 3D printed-electrospun biphasic PCL/nHA/MWCNTs scaffold with disordered nanospun fibers above and porous microscale layer below. Reproduced with permission from (Cao et al., 2023). **(C)** 3D printed biphasic porous scaffold with its cartilage and subchondral layers are made of AS and APS, respectively. Reproduced with permission from (Joshi et al., 2022). **(D)** 3D printed biphasic porous scaffold with its cartilage and subchondral layers are made of peptide-GelMA and HAp/GelMA, respectively. Reproduced with permission from (Ding et al., 2022). **(E)** The biphasic scaffold included the upper GelMA layer prepared by DLP with a radially oriented structure and the lower PCL-HA layer prepared by FDM with 0°, 90° log pile porous structure. Reproduced with permission from (Gong et al., 2020). **(F)** Schematic illustration of preparation of 3D printed biphasic scaffold. PCL was first extruded to print outline and DBM/SF bioink was printed to fill the space to fabricate the bone layer. DCM/SF bioink was used to print the cartilage layer on the bone layer. Reproduced with permission from (Zhang et al., 2021).

Biphasic scaffolds include bone and cartilage phases. The structure and biomechanical properties of the lower bone phases are similar to normal bone tissues. The upper cartilage phases mainly play the role of temporary ECM. Based on the differences in material composition and structural design, there are three main scaffold designs for biphasic scaffolds: biphasic scaffolds with same material composition ratio but different structural design (SM/DS-biphasic scaffolds), biphasic scaffolds with different material composition ratio but same structural design (DM/SS-biphasic scaffolds), and biphasic scaffolds with different material composition ratio and different structural design (DM/DS-biphasic scaffolds) (Table 3). The characteristics of the above three types of biphasic scaffolds are shown in Figure 2. The design of biphasic scaffolds is more complex than that of monophasic scaffolds. In addition to the differences in material composition ratio and structural design between the upper and lower layers, it also involves the addition of different cells and growth factors between the two layers, to obtain a more definite purpose of osteogenesis and cartilage formation.

**TABLE 3:** 3D printed osteochondral biphasic scaffolds.

Types	Subchondral bone phase		Cartilage phase		Printing mode	*in vivo*/*in vitro*	Ref
	Materials	Structure	Materials	Structure			
SM/DS- biphasic scaffolds	PCL	Pore size 400 μm	PCL	Pore size 200 μm	FDM	Both	Wang et al. (2022b)
	PCL; nHA; MWCNTs	0°, 90° log pile	PCL; nHA; MWCNTs	Disordered nanospun fibers	EP/ES	*In vitro*	Cao et al. (2023)
DM/SS- biphasic scaffolds	PLA; G5	0°, 90° log pile	PLA	0°, 90° log pile	EP	Both	Barbeck et al. (2017)
	GelMA; PEGDA; nHA	0°, 90° log pile	GelMA; PEGDA; PLGA-NPs	0°, 90° log pile	EP	*In vitro*	Zhou et al. (2019)
	PACG; GelMA; BAG	0°, 90° log pile	PACG; GelMA; Mn^2+^	0°, 90° log pile	EP	Both	Gao et al. (2019)
	CPC	0°, 90° log pile	ALGMC	0°, 90° log pile	EP	*In vitro*	Kilian et al. (2020)
	GelMA; HA	0°, 90° log pile	GelMA	0°, 90° log pile	EP	Both	Gao et al. (2021a)
	GM; SF-MA	0°, 90° log pile	GM; SF-PTH	0°, 90° log pile	EP	Both	Deng et al. (2021)
	Cellulose; BG	Nanofiber structure	Cellulose	Nanofiber structure	EP	Both	Guo et al. (2022)
	GelMA; HA	0°, 90° log pile	Peptide-GelMA	0°, 90° log pile	EP	Both	Ding et al. (2022)
	HA; γ-PGA	0°, 90° log pile	Col; γ-PGA	0°, 90° log pile	EP	Both	Nguyen et al. (2022)
	Alginate PO_4_; SF; GEL	0°, 90° log pile	Alginate; SF; GEL	0°, 90° log pile	EP	*In vitro*	Joshi et al. (2022)
DM/DS- biphasic scaffolds	PCL; HA	0°, 90° log pile	GelMA	Radially oriented Structure	FDM/DLP	Both	Gong et al. (2020)
	PCL; PLGA; β-TCP	Honeycomb pattern	PCL; PLGA; CS	Cubical pattern	EP	Both	Natarajan et al. (2021)
	PCL; DBM; SF	Strengthened framework with 0°, 90° log pile	DCM; SF	0°, 90° log pile	EP	*In vitro*	Zhang et al. (2021)

Abbreviations: FDM, fused deposition modeling; EP, extrusion printing; ES, electrospinning; DLP, digital light processing; MWCNTs, multi-walled carbon nanotubes; nHA, nano-hydroxyapatite; G5, calcium phosphate-based glasses (P2O5-CaO-Na2O-TiO2); PLGA-NPs, PLGA-nanoparticles; PACG, poly (N-acryloyl 2-glycine); CPC, calcium phosphate cement; ALGMC, alginate-methylcellulose; GM, gelatin methacryloyl; SF-MA, silk fibroin with methacrylic anhydride; SF-PTH, silk fibroin with parathyroid hormone; DBM, bone decellularized extracellular matrix; DCM, cartilage decellularized extracellular matrix.

Biphasic scaffolds have different designs of upper and lower structures, so the same or different printing methods can be used. The preparation of SM/DS- biphasic scaffolds by 3D printing is rarely reported in OC tissue applications. It may be that the change of scaffold structure alone cannot fully utilize the bidirectional effect of osteogenesis and chondrogenesis (Wang Y. et al., 2022; Cao et al., 2023). Cao et al., 2023 successfully constructed PCL/nano-hydroxyapatites/multi-walled carbon nanotubes (PCL/nHA/MWCNTs) biphasic scaffolds by the combination of electrospinning and layer-by-layer 3D printing. This dual-scale scaffold consisted of a dense layer of disordered nanospun fibers and a porous microscale 3D scaffold layer to improve the osteogenic differentiation of BMSCs *in vitro*. However, there is a lack of verification *in vivo* studies. Wang Y. et al., 2022 prepared a composite scaffold (BE-PSA) with gradient structure and programmed biomolecule delivery by FDM 3D printing and multi-material-based modification. The 3D printed PCL scaffold included upper pores of 200 μm for cartilage regeneration and lower pores of 400 μm for bone regeneration. This structural difference in the pore size of the upper and lower layers structurally mimicked the gradient structure of OC tissue, but didn’t have a biphasic induction. Therefore, for a sequential modulation of BMSCs behavior, E7 peptide and B2A peptide were added to the scaffold to improve the migration and osteogenic/chondrogenic differentiation of BMSCs. In recent years, a series of DM/DS-biphasic scaffolds have been constructed by adding osteogenic and chondrogenic components to the upper and lower phases of SM/DS-biphasic scaffolds, respectively. These biphasic scaffolds often incorporate bone matrix ceramic-like materials in the subchondral bone phase and cartilage matrix-like hydrogel materials in the cartilage phase (Gong et al., 2020; Natarajan et al., 2021; Zhang et al., 2021). For example, Gong et al. developed a biphasic scaffold with the GelMA scaffold printed by DLP in the upper layer and with the PCL/HA scaffold printed by FDM in the lower layer (Gong et al., 2020). Amrita et al. devised the strategy of 3D printing for fabricating biphasic PCL/PLGA scaffolds that are loaded with bioactive factors (chondroitin sulphate and beta-tricalcium phosphate (β-TCP)) for the upper cartilage and lower bone layer respectively (Natarajan et al., 2021). Zhang et al., 2021 fabricated 3D printed bilayered constructs. PCL was first extruded to print the frame of the bone layer, and the bone decellularized extracellular matrix (DBM) bioink was printed to fill the space. The cartilage decellularized extracellular matrix (DCM) bioink was used to print the cartilage layer on the bone layer. Finally, the scaffold was added TGF-β1 and BMP-2 to promote OC regeneration.

DM/SS- biphasic scaffolds have been studied more extensively. Initially, not all such biphasic scaffolds were based on 3D printing integrated molding, but were prepared by a combination of printing technology and conventional preparation methods. Yang T. et al., 2021 developed a bilayered OC scaffold. The subchondral bony compartment was prepared from 3D printed Ti alloy, and the cartilage compartment was created from a freeze-dried collagen sponge, which was reinforced by PLGA. Mechanical support provided by 3D printing Ti alloy promotes cartilage regeneration by promoting subchondral bone regeneration and providing a mechanical support platform for cartilage synergistically. However, the scaffold is not integrally molded, the preparation process is relatively complicated, and the interface bonding is not stable enough. Therefore, integrated scaffolds based entirely on 3D printing are the focus of current research. Therefore, the integrated stent based entirely on 3D printing is the focus of current research. The upper and lower scaffolds often use the same printing method. Meanwhile, material selection and structural design are more extensive (Barbeck et al., 2017; Aisenbrey et al., 2018; Gao et al., 2019; Kilian et al., 2020; Gao J. et al., 2021; Deng et al., 2021; Ding et al., 2022; Guo et al., 2022; Joshi et al., 2022; Nguyen et al., 2022). Since the molding method of EP is relatively mild and can print cell-loaded bioinks, most researchers use EP to construct DM/SS-biphasic scaffolds with porous structures on the upper and lower layers. The 3D printed scaffolds were confined to a 0, 90° log pile design that could have been designed or a limitation of the printing technique in combination with the materials. Gao et al., 2019 successfully constructed a biodegradable high-strength supramolecular polymer-strengthened hydrogel composed of cleavable poly (N-acryloyl 2-glycine) (PACG) and GelMA (PACG-GelMA) by photo-initiated polymerization. Then, a biohybrid gradient scaffold consisting of the top layer of PACG-GelMA hydrogel-Mn^2+^ and the bottom layer of PACG-GelMA hydrogel-bioactive glass is fabricated for repair of OC defects by a 3D printing technique. This scaffold not only enhanced chondrogenic-related and osteogenic-related differentiation of BMSCs, but also facilitated concurrent regeneration of cartilage and subchondral bone in a rat model.

Compared with monophasic scaffolds, biphasic scaffolds provide suitable microenvironments for cartilage and bone regeneration in terms of mimicking native tissue architecture, respectively. However, there may be a problem of poor bonding at the OC interface, and the separation of the bonding site may occur in in vivo studies, resulting in unsatisfactory repair effects.

### 5.3 Triphasic scaffolds

Based on the biphasic scaffolds, the triphasic scaffolds add an intermediate layer between the bone layer and the cartilage layer to simulate the calcified layer of natural cartilage. The calcified layer is a narrow, highly mineralized zone that marks the transition from soft cartilage to stiff subchondral bone and has the function of distributing transverse stress and resisting shear forces (Simkin, 2012; Goldring and Goldring, 2016). The dense calcified layer divides the OC tissue into two different microenvironments, which limits the free exchange of interstitial fluid between subchondral bone and cartilage. Therefore, the introduction of the transition layer not only serves as a physical barrier to inhibit blood vessels from invading cartilage and prevent full-thickness cartilage ossification, but also supports the load of articular cartilage, which is beneficial to the fusion of the implant and the host tissue at the interface (Levingstone et al., 2014). The triphasic scaffold is usually made of a variety of materials, which can better simulate the structural distribution of normal tissues, so it can simultaneously reconstruct cartilage, calcified layer, and subchondral bone, and realize the integration of OC repair.

The construction of the triphasic scaffold is more complicated, involving the material components selection, internal structural design, and mechanical attribute differences of each layer (Zhang T. et al., 2017; Li Z. et al., 2018; Zhu M. et al., 2020; Diloksumpan et al., 2020; Mellor et al., 2020; Irmak and Gümüşderelioğlu, 2021; Qiao et al., 2021; Wang et al., 2023), as shown in Table 4. Generally, the triphasic scaffold adopts a combination of 3D printing technology and traditional methods to prepare the scaffold of each layer. As for the bonding of each layer, it is also continuously developing (Nooeaid et al., 2012; Zhang et al., 2020b). Initially, the binding method used a solvent to slightly soluble the ends of the scaffolds, and then the ends of the two scaffolds were gently pressed together for adhesion. Li Z. et al., 2018 designed a multilayer composite scaffold containing cartilage, bone, and calcified layers to simulate physiological full-thickness bone-cartilage structure. The bone and calcified layers were synthesized with PLGA and β-TCP composite using a low-temperature 3D bioprinter. The cartilage layer was created with a cartilage matrix from bovine articular cartilage using an improved temperature-gradient thermally induced crystallization technology. Then, he used the “lysis-adhesion technique” to fix the three layers together to obtain a stable triphasic bionic scaffold. With the development of material modification, the bonding method is also changing, and the layers can be bonded to each other by immersion in modified hydrogels and UV cross-linking. Qiao et al., 2021 produced a bioinspired tri-layered fiber-hydrogel construct via MEW and FDM techniques. The fibrous scaffolds were gradually immersed in GelMA hydrogel solution, crosslinking was accomplished by UV irradiation. The three-layer composite scaffold is made by a three-step crosslinking program. In addition, some studies have been devoted to the development of hydrogel materials suitable for different layers. Because the hydrogel has a certain viscosity, it can directly construct integrated bionic three-phase scaffolds through 3D printing technology. The layers of the scaffolds are directly bonded to each other without the need for other bonding methods (Irmak and Gümüşderelioğlu, 2021). Zhu M. et al., 2020 developed a triphasic scaffold with enhanced interface bonding through 3D printing. The one-shot printing process enabled control over material composition, pore structure, and size in each region of the scaffold while realizing a seamlessly integrated construct as well. The scaffold was designed to be triphasic: a porous bone layer composed of alginate sodium (SA) and mesoporous bioactive glasses (MBG), an intermediate dense layer also composed of SA and MBG, and a cartilaginous layer composed of SA.

**TABLE 4:** 3D printed osteochondral triphasic scaffolds.

	Subchondral bone phase	Calcified cartilage phase	Cartilage phase	Ref
Material	PLGA, TCP, type I collagen	PLGA, TCP, type I collagen	Cartilage ECM, chitosan	Zhang et al. (2017a)
Structure	0°, 90° log pile	compact structure	Orientated casted hydrogel	
Fabrication technique	LDM	LDM	TIPS	
Cells	None	None	None	
Factors	None	None	None	
Material	PLGA, β-TCP	PLGA, β-TCP	Cartilage ECM	Li et al. (2018c)
Structure	0°, 90° log pile	compact structure	Orientated casted hydrogel	
Fabrication technique	LDM	LDM	TIPS	
Cells	None	None	None	
Factors	None	None	None	
Material	α-TCP, nHA, Poloxamer	PCL + all materials in bone/cartilage phase	PCL, GelMA	Diloksumpan et al. (2020)
Structure	0°, 0°, 90°, 90° log pile	0°, 0°, 90°, 90° log pile; 0°, 90° log pile	0°, 90° log pile infiltrated with casted GelMA	
Fabrication technique	EP	MEW, EP, Casting	MEW, Casting	
Cells	None	None	None	
Factors	None	None	None	
Material	PCL, β-TCP	PCL	PCL, dECM	Mellor et al. (2020)
Structure	0°, 120°, 240° log pile	electrospun PCL disc	0°, 120°, 240° log pile	
Fabrication technique	EP	ES	EP, Casting	
Cells	None	None	None	
Factors	None	None	None	
Material	SA, MBG	SA, MBG	SA	Zhu et al. (2020a)
Structure	0°, 60° log pile	compact structure	0°, 90° log pile	
Fabrication technique	EP	EP	EP	
Cells	None	None	None	
Factors	None	None	None	
Material	PCEC, nHA, GelMA	PCEC, GelMA	PCEC, GelMA	Qiao et al. (2021)
Structure	0°, 90° log pile infiltrated with casted GelMA	0°, 90° log pile infiltrated with casted GelMA	0°, 30° log pile infiltrated with casted GelMA	
Fabrication technique	FDW, Casting	MEW, Casting	MEW, Casting	
Cells	BMSCs	BMSCs	BMSCs	
Factors	BMP-2	TGF-β1	BMP-7, TGF-β1	
Material	GelMA	GelMA, PRP (1:0.5)	GelMA, PRP (1:1)	Irmak and Gümüşderelioğlu (2021)
Structure	Compact structure	Compact structure	Compact structure	
Fabrication technique	EP	EP	EP	
Cells	AdMSCs	AdMSCs	AdMSCs	
Factors	None	PDGF, TGF-β, bFGF	PDGF, TGF-β, bFGF	
Material	CSi-Mg6	PLCL, BG, CS	PLGA, fibrin	Wang et al. (2023)
Structure	Pore size 380 μm	Fiber structure	Pore size 280–450 μm with casted fibrin	
Fabrication technique	DLP	ES	Gelatin porogen-leaching method, Casting	
Cells	None	None	BMSCs	
Factors	None	None	TGF-β1	

Abbreviations: LDM, low-temperature deposition; TIPS, thermal-induced phase-separation; EP, extrusion printing; MEW, melt electro-writing; ES, electrospinning; FDW, fused deposition modeling; DLP, digital light processing; SA, alginate sodium; MBG, mesoporous bioactive glasses; PCEC, poly (ε-caprolactone) and poly (ethylene glycol); PRP, platelet-rich plasma; PDGF, growth factors; AdMSCs, adipose tissue-derived mesenchymal stem cells.

**FIGURE 3:**
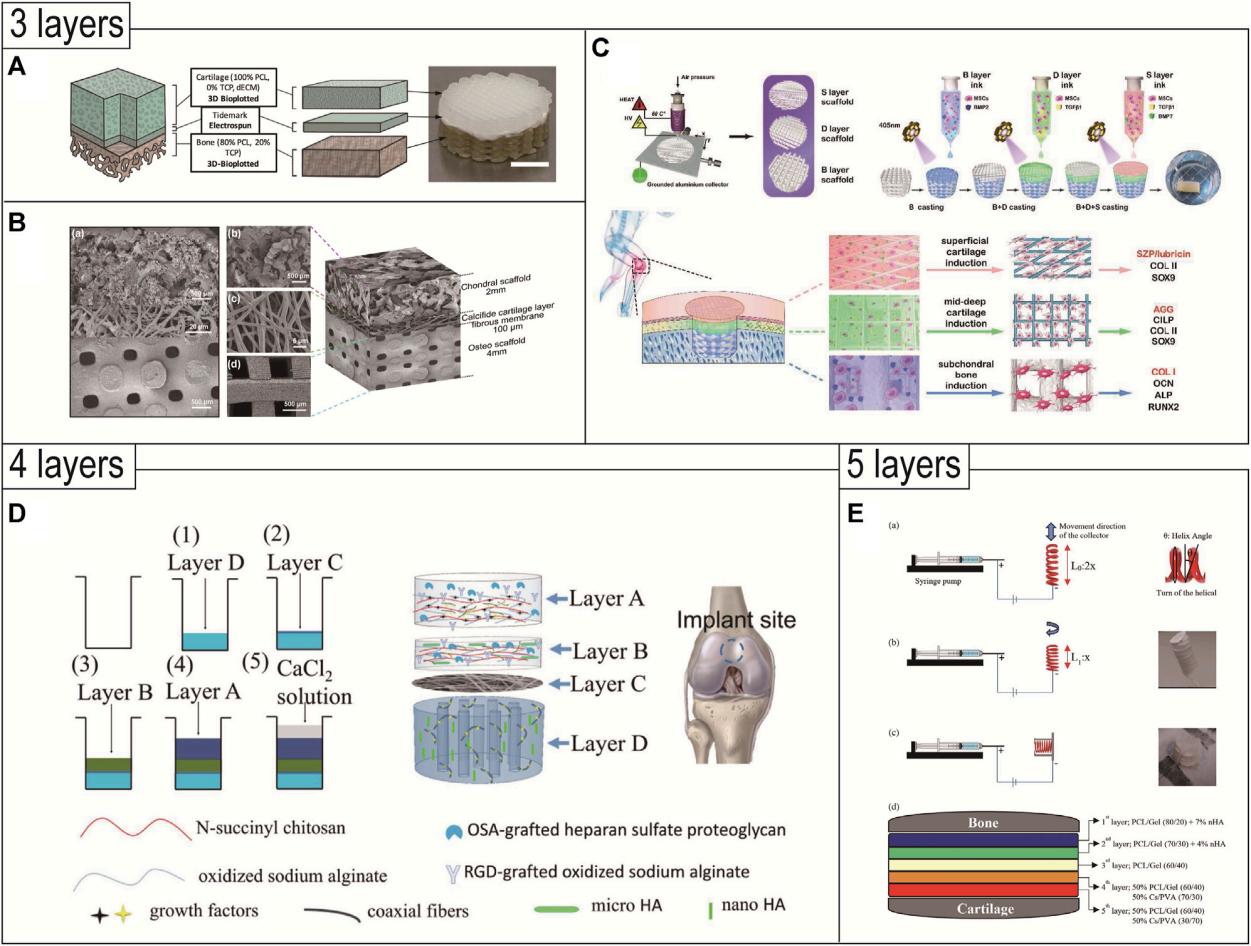
3D printed triphasic **(A–C)**/multiphasic **(D** and **E)** scaffolds for OC tissue engineering. **(A)** Triphasic scaffolds comprised of 3D-bioplotted PCL-TCP (bone layer), electrospun PCL (tidemark), and 3D-bioplotted PCL-dECM (cartilage layers) phases were evaluated and demonstrated site-specific OC tissue characteristics. Reproduced with permission from (Mellor et al., 2020). **(B)** Triphasic scaffolds comprised of a PLGA scaffold loaded with fibrin hydrogel, BMSCs, and TGF-β1 for cartilage tissue, an electrospun PLCL-fibrous membrane loaded with chondroitin sulfate, and bioactive glass for calcified cartilage, and a 3D printed calcium silicate ceramic scaffold for subchondral bone. Reproduced with permission from (Wang et al., 2023). **(C)** A tri-layered (superficial cartilage (S), deep cartilage **(D)**, and subchondral bone **(B)** layer) stratified scaffold in which a MSC-laden GelMA hydrogel with zone-specific growth factor delivery was combined with melt electrowritten triblock polymer of poly (ε-caprolactone) and poly (ethylene glycol) (PCEC) networks with depth-dependent fiber organization. Reproduced with permission from (Qiao et al., 2021). **(D)** Based on composite materials of SA, chitosan, and HAp with different micro and nano sizes, an intermediate calcified cartilage layer as well as a subjacent electrospun fiber membrane for cell migration prevention was designed in the four-layered scaffold to repair OC defects. Reproduced with permission from (Chen T. et al., 2018). **(E)** The novel 3D-functionality graded nanofibrous scaffolds composed of five layers based on different compositions containing PCL/gelatin (Gel)/nanohydroxyapatite (nHA) for osteoregeneration and chitosan (CS)/polyvinylalcohol (PVA) for chondral regeneration are fabricated by electrospinning technique. Reproduced with permission from (Mancini et al., 2020).

The triphasic scaffolds can better simulate the distribution of normal tissue structure, which is more in line with the requirements of tissue-engineered OC composite scaffolds (Figures 3A–C). It is worth noting that the bonding strength of the scaffold’s adjacent interfaces needs to be strengthened, and the design simulation of the tideline as well as the calcified cartilage layer is still immature. The composition of the triphasic scaffold structure, the optimization of parameters, the selection of materials, and the validation of *in vivo* and *ex vivo* experiments are also some of the research content in the field of organizational engineering in the future.

### 5.4 Multiphasic scaffolds

The design of multiphasic scaffolds is the most complex, including at least four or more different layers in the OC scaffold (Figures 3D,E). This shift from triphasic scaffolds to multiphasic scaffolds usually takes into account the structure of the different regions of the cartilage tissue and is more conducive to smooth transitions between the different layers of the OC tissue. Since it is more difficult to realize such complex designs, existing studies often combine 3D printing technology with traditional methods to prepare such multiphasic scaffolds. Chen et al. prepared a multiphasic OC scaffold consisting of four different layers. The modified natural hydrogel was doped with various growth factors and HA particles to produce the cartilage phase, calcified cartilage phase, and bone phase. PCL/PEG electrospun fiber membrane was located above the bone phase, which is used to prevent the migration of cells in the upper and lower layers and to restrict the spatial distribution of cells. The scaffold showed a good effect of OC repair in rabbits after 12 weeks. The scaffold was well combined with the cartilage and bone cavity of the host tissue, and regenerated cartilage thickness matched that of the host tissue (Chen T. et al., 2018). Mancini et al. proposed a four-phase OC scaffold that better mimics the regional structure of articular cartilage. The PCL mesh scaffold with a 0°, 90° log pile pattern served as the base. The PCL scaffold porosity gradually decreased from the subchondral bone layer to the calcification layer, until the texture was dense and served as the interface region. Then the PCL was compounded with hydrogel containing MSC and printed into a scaffold with 70% porosity as the subchondral layer. Next, the PCL was removed, leaving only the hydrogel and articular cartilage progenitor cells (ACPCs) as the cartilage surface layer. The scaffold adequately mimicked the gradient layered structure of OC tissue and was more conducive to OC repair (Mancini et al., 2020).

Hejazi et al., 2021 developed novel 3D-functionally graded nanofibrous scaffolds composed of five layers using ES. The five-phase scaffold contained PCL/gelatin/nanohydroxyapatite for osteoregeneration and chitosan/polyvinylalcohol for chondral regeneration. In this design, each layer had a fibrous structure with continuous nanofibers, and the pore size and porosity of the novel 3D scaffold were improved, however, with no full biological characterization of the scaffold, it remained to be seen whether the scaffold with five phases has an advantage over those with 2–4 phases.

## 6 Continuous gradient scaffolds

To successfully construct a scaffold that conforms to the natural structure of OC tissue, it is necessary to simulate the physiological properties of natural OC tissue as closely as possible. In the existing studies, OC gradient scaffolds can be divided into discrete gradient scaffolds and continuous gradient scaffolds (Zhang et al., 2020b). Discrete gradient scaffolds include biphasic, triphasic and multiphasic scaffolds, each phase represents a specific region of OC organization, and these scaffolds have been described in detail in the previous sections (Section 5.2, 5.3, and 5.4). Continuous gradient scaffolds have a gradual transition and no distinct interface between each layer which more closely mimic the original characteristics of the OC tissue. Continuous gradient scaffolds don’t exhibit individual layers and are constructed as whole scaffolds with gradient characteristics. Compared with discrete gradient scaffolds, continuous gradient scaffolds better simulate the compositional and structural transitions in native OC tissue and minimize shear stresses between adjacent regions (Lowen and Leach, 2020). Therefore, continuous gradient scaffolds have the potential to induce smooth transitions between components of OC tissue, reducing interface instability.

Material selection and structural building are interconnected and codependent steps of the OC scaffold design process. Therefore, the fabrication of continuous gradient scaffolds requires not only suitable biomaterials, but also suitable manufacturing methods. Manufacturing methods can be divided into conventional methods, 3D printing, and emerging technologies. Conventional methods, including solvent-casting (Petit et al., 2019), gas-molding (Loh and Choong, 2013) and freeze-drying (Pitrolino et al., 2022), have high-cost performance, and can mainly control the gradient of the microstructure (including pore size and porosity) of the scaffold to a certain extent (Frassica and Grunlan, 2020). Emerging technologies include buoyancy, magnetic attraction and electrical attraction technologies, which are usually combined with specific materials and mainly gradient control of the distribution of material components of the scaffold (Li C. et al., 2018; Li et al., 2019; Xu et al., 2020). However, these two kinds of methods are flexible in the gradient distribution of the scaffold, and it is difficult to achieve precise control at the microscopic level. However, 3D printing technology can independently regulate the macro and micro characteristics of the scaffold, which can further develop continuous gradient scaffold with specific structures (Bittner et al., 2019). In general, continuous gradient scaffolds produced by 3D printing include gradient material, gradient structure, the both of gradient material and structure, and gradient interface. The articles reviewed below are limited to continuous gradient scaffolds fabricated by using 3D printing for the repair of OC defects, and exclude those developed by using conventional methods or emerging technologies alone. Moreover, we have summarized the most recent studies about 3D printed OC continuous gradient scaffolds, as shown in Table 5.

**TABLE 5:** 3D printed osteochondral continuous gradient scaffolds.

Established gradients	Fabrication technique	Gradient characteristics	*in vivo* */* *in vitro*	Ref
Gradient material	Selective laser sintering (SLS)	From the upper cartilage layer to the lower bone layer, HAp content was increased from 0% to 30% with 5% increments	both	Du et al. (2017)
Gradient structure	Extrusion printing (EP)	PCL (pore sizes ranged from 150 to 750μm, from the top to the bottom) continuous gradient structures to enhance a hydrogel scaffold	both	Sun et al. (2020)
Gradient structure	Extrusion printing (EP)	3D-printed PCL based osteal anchor (porosity from top to bottom of 70%, 0%, 25%, 30%, 35%, 40%) for fixation of reinforced hydrogels	both	Mancini et al. (2017)
Gradient structure	Bioprinting	PLA gradient scaffold with porosity ranging from 30% to 60% in increments of 5%, from top to bottom	*in vitro*	Golebiowska and Nukavarapu (2022)
Gradient structure	Direct ink writing (DIW)	PCL/HAp gradient scaffold with pore angle ranging from 90° to 15°, from top to bottom	*in vitro*	Zhang et al. (2020a)
Gradient material and structure	Inkjet printing (IP)	The gradient scaffold with both gradient characteristics of the composition (PCL combined with 0, 15 to 30 wt% HAP) and the microstructure (pore size from top to bottom of 200,500, and 900 μm, respectively)	*in vitro*	Bittner et al. (2019)
Gradient material and structure	Hybrid twin-screw extrusion/electrospinning (TSEE)	Functionally graded non-woven meshes of polycaprolactone incorporated with tricalcium phosphate nanoparticles	*in vitro*	Erisken et al. (2008)
Gradient interface	Aspiration-extrusion printing method	A gradient hybrid interface with the different aspirating methods of the CMCTyr-Gel and Alg-Tyr-Gel inks materials	*in vitro*	Senturk et al. (2023)
Gradient interface	Combined EP with microfluidics	The deposition of continuous gradients of chemical, mechanical, and biological signals, as well as the fabrication of scaffolds with very high shape fidelity and cell viability	both	Idaszek et al. (2019)

### 6.1 Gradient material

In the construction of continuous gradient scaffolds, creating material composition gradient is a common strategy to mimic OC structure characteristics. The proportions and types of collagen and hydroxyapatite that give the strength and hardness of the osteochondral tissue change and produce different cellular environments (Amann et al., 2021; Cao et al., 2022). In order to generate material composition gradients, most studies have used conventional methods to construct scaffolds using HAP particles in combination with PCL or gelatin. Cristian et al. used a sequential addition technique to combine all the four composite aqueous suspensions to fabricate a gradient scaffold. The scaffold started from the slurry with the highest HAp content, the HAp/Coll 50/50 slurry, followed by a decreasing HAp content up to the pure collagen slurry (HAp/Coll 0/100), which demonstrated positive impacts on chondrogenic and osteogenic differentiation, tissue regeneration and/or improvement of mechanical properties (Parisi et al., 2020). Recent studies have combined 3D printing technology into OC continuous gradient scaffolds, which can prepare a continuous scaffold with more component gradient levels. A seven-layered gradient scaffold consisting of basic building blocks of PCL and HAp microspheres was prepared through SLS. From the upper cartilage layer to the lower bone layer, HAp content was increased from 0% to 30% with 5% increments. The results of *in vitro* cellular evaluation and *in vivo* implantation results confirmed its capability in inducing the formation of cartilage and subchondral bone tissues (Du et al., 2017). Although continuous gradient scaffolds with only material gradient have several advantages, they still need to be further adjusted to achieve the desired overall performance.

### 6.2 Gradient structure

The gradient microstructure in OC tissue is interconnected and plays an important role in nutrient and oxygen transportation, cell adhesion and migration, and vascular ingrowth (Wang M. O. et al., 2015; Perez and Mestres, 2016). Smaller pores (<0.1–0.3 mm) tend to restrict nutrient transportation, vessel formation, and contribute to the formation of new cartilage (Temenoff and Mikos, 2000). While larger pores (>0.3 mm) will promote the transport of nutrients, bone cell migration, and more suitable for subchondral bone repair (Takahashi and Tabata, 2004; Duan et al., 2014). In 3D printed continuous gradient scaffolds, gradient construction of the microstructure is usually achieved by continuously changing the pore size (Sun et al., 2020; Gregory et al., 2023), porosity (Mancini et al., 2017; Golebiowska and Nukavarapu, 2022), and pore angle (Zhang et al., 2020a). In these studies, Sun et al., 2020 printed PCL continuous gradient structures to enhance a hydrogel scaffold containing MSCs, where gradient pore sizes ranged from 150 to 750 μm and gradient microchannels of PCL gradually widened downward from the surface cartilage region. The scaffold provided suitable mechanical properties and excellent cell differentiation, which further promoted the formation of new cartilage and vessels ingrowth in the bone layer. In terms of porosity, 3D printing usually combines polymers (PCL, PLA, etc.), and the porosity increases from 30% to 70% according to the characteristics of OC tissue structure, which is more conducive to the smooth transition between bone and cartilage (Mancini et al., 2017; Golebiowska and Nukavarapu, 2022). Combined with the characteristics of 3D printing, the pore angle of continuous gradient was set. (Zhang et al., 2020a) used direct ink writing to construct a continuous gradient scaffold, and the laying angle was reduced from 90° to 15°. By using finite element simulation to adjust the porosity and local placement angle in the printed scaffold, the layered mechanical properties of natural OC tissue could be simulated. However, microstructural changes alone do not have a significant effect (Barron et al., 2016), whereas biological additives such as growth factors seem to have a greater impact on the outcome of OC regeneration (Zhang Y. T. et al., 2017). Therefore, continuous gradient scaffolds with both material and structural gradients may provide a better solution.

### 6.3 Gradient material and structure

Polymers (such as PCL) are mostly heat sensitive materials, which are more suitable for 3D printing technology and can prepare high precision scaffolds. Bittner et al. manufactured scaffolds with high fidelity and gradient distribution of composition and porosity. The scaffold fabricated by EP to simulate the both gradient characteristics of the composition (PCL combined with 0, 15 to 30 wt% HAP) and the microstructure (pore size from top to bottom of 200,500, and 900 μm, respectively) of OC tissue. They observed that the mechanical properties of this double (porosity and composition) gradient scaffold were similar to those of the homogeneous scaffold with the highest porosity, with the potential to better match natural tissue physiology and promote tissue regeneration (Bittner et al., 2019). Cevat et al. demonstrated the fabrication and utilization of functionally graded non-woven meshes of polycaprolactone incorporated with tricalcium phosphate nanoparticles using a new hybrid twin-screw extrusion/electrospinning (TSEE) process, allowing the generation of continuous spatial gradations in composition and porosity of electrospun nanofibrous membranes. The scaffold was seeded and cultured with mouse preosteoblast cells (MC3T3-E1). Within 4 weeks, the tissue constructs revealed the formation of continuous gradients in the extracellular matrix with various markers, including collagen synthesis and mineralization, similar to the type of variations observed in the typical bone-cartilage interface in terms of the distributions of concentration of Ca particles and of mechanical properties associated with this (Erisken et al., 2008). However, there are few studies on 3D printed scaffolds with continuous gradient of composition and structure, and there are no relevant *in vivo* studies. Whether such scaffolds are better than scaffolds with continuous gradient of composition or structure is unknown, and further studies are still needed.

### 6.4 Gradient interface

Researches in recent years have combined 3D printing technology with other techniques aimed at preparing scaffolds with a continuous gradient distribution of material and/or structure at the interface (Idaszek et al., 2019; Senturk et al., 2023; Wei et al., 2023). The interface of this kind scaffold is usually formed by the fusion of cartilage layer and bone layer material, so the binding force at the interface junction is relatively strong. Efsun et al. combined 3D bioprinting and an aspiration-extrusion microcapillary method to fabricate hydrogel scaffolds. Different hydrogels containing cells were sucked into the same microcapillary glass, and then the hydrogels were printed into OC scaffolds with precise gradient components using microcapillary bioprinting. *In vitro* experiments had shown that the scaffolds successfully induce the differentiation of MSC into chondrogenic and osteoblastic tissues with controlled interfaces (Senturk et al., 2023). Joanna et al. combined EP with microfluidics, using a mixed doped alginate-based solution. The biomaterials mimicked the zonal cartilage organization and extracellular matrix composition by using a microfluidic printing head with a mixing unit and incorporated into an extrude-based printer. The technology facilitated the deposition of continuous gradients of chemical, mechanical, and biological signals, as well as the fabrication of scaffolds with very high shape fidelity and cell viability (Idaszek et al., 2019). However, these scaffolds are limited by manufacturing technology and often used hydrogel materials with high fluidity, so the mechanical properties of the scaffolds are week and do not provide precise control over pore size, microstructure, and pore interconnections. However, its biggest advantage lies in the smooth transition of the interface and the strong binding force.

## 7 Seed cells in OC tissue engineering

In order to restore the structure and function of OC defects, the addition of specific cells to scaffolds is widely used in the field of OC tissue engineering. The incorporated cells can improve ECM deposition and tissue regeneration, promote the interaction between the scaffold and the surrounding host tissue, and thus affect the way the overall healing occurs. Therefore, the design of biomimetic OC tissue requires the selection of suitable osseous and cartilaginous sources that meet specific criteria (Nooeaid et al., 2012; Nukavarapu and Dorcemus, 2013). The cell sources commonly used in OC tissue engineering scaffolds include primary cells and stem cells. Chondrocytes and osteoblasts are widely used in primary cells. Stem cells are divided into pluripotent stem cells and adult stem cells. Among them, pluripotent stem cells include embryonic stem cells (ESCs) and induced pluripotent stem cells (iPSCs), and adult stem cells mostly refer to bone marrow-derived stem cells (BMSCs) and adipose-derived stem cells (ASCs) (Gonçalves et al., 2021).

In 3D printed OC scaffolds, primary cells (chondrocytes and osteoblasts) are used exclusively for cartilage and subchondral bone regions, and stem cells are often used for the entire region of the scaffold. The cells used may be allogeneic or allogeneic (Canadas et al., 2018; Maia et al., 2018; Nam et al., 2018; Luby et al., 2019). Although pluripotent stem cells have a highly differentiated and self-renewing ability to differentiate into osteogenic and chondroblast lineages, they are rarely used in 3D printed OC scaffolds, which may be due to the difficulty in controlling the degree of cell differentiation and tumorigenic potential *in vivo* (Sundelacruz and Kaplan, 2009; Ko et al., 2014). Chondrocytes in the process of culture tend to dedifferentiate, minimally producing collagen II, which is characteristic of hyaline cartilage, but producing collagen I instead (Charlier et al., 2019; Yao and Wang, 2020). Osteoblasts are easy to cause damage and infection at the donor site during isolation, and have low proliferation potential and limited number of cells obtained (Panseri et al., 2012). As a result, there are less and less researches on the inclusion of primary cells into 3D printed OC scaffolds. In recent years, the researches of adult stem cells in OC tissue engineering have become more and more extensive. In particular, BMSCs, derived from bone marrow, are relatively easy to separate and proliferate, and can differentiate into chondrocytes and osteoblasts (Gao Q. et al., 2021). Combined with 3D printing technology, microenvironments conducive to chondrogenesis and osteogenesis can be customized in different regions of the scaffold. In addition, ASCs can be isolated from subcutaneous adipose tissue using minimally invasive methods, thus avoiding donor site morbidity and patient pain while providing a large number of cells, making it a possible choice for OC tissue engineering (Zhang K. et al., 2016).

In the process of 3D printing the OC scaffold, MSCs can be mixed into the printing ink and printed at the same time. Alternatively, MSCs are inoculated on printed OC scaffolds. The choice of the two methods depends on the properties of the printing ink, such as fluidity, softness and hardness, and the survival rate of the cells in it. Regardless of the loading method, due to the bidirectional induction of MSCs, inducers such as growth factors that promote differentiation are generally added to the cartilage layer and bone layer of scaffolds, respectively. Zhang et al. developed a bioink composed of acellular extracellular matrix and fibroin protein to print a double-layer scaffold that encapsulated transforming growth factor-β (TGF-β) and bone morphogenetic protein-2 (BMP-2) as a controlled release system, promoting OC regeneration in a rabbit knee joint model (Zhang et al., 2021). In addition to incorporating this conventional inducible growth factor to the scaffold, polypeptides can also be added to promote the migration and differentiation of MSCs (Wang Y. et al., 2022). With the continuous deepening of relevant researches, the loading method of cell balls has also been incorporated into the 3D printed OC scaffold. Adipose-derived stem cells (hADSCs) and nanofibers were coated with TGF-β3 or BMP-2 for chondrogenesis or osteogenesis, respectively, and both types of composite spheres were prepared. Each type of spheroid was then cultured within a 3D printed microchamber in a spatially arranged manner to recapitulate the bilayer structure of osteochondral tissue (Lee et al., 2022). The combination of cells, factors, and printing technology will continue to build more complete OC scaffolds, opening up new opportunities for OC defect.

## 8 Discussion and future outlooks

OC defects are a widespread and serious osteoarticular disease in clinical practice. Effective repair of OC defects has been an urgent challenge in the field of tissue engineering. In this paper, the research progress of 3D printed OC tissue engineering scaffolds is discussed in detail based on the gradient structure and biological properties of articular OC tissue. With the development of 3D printing technology and a deeper understanding of OC structure, researchers have begun to consider the feasibility of applying 3D printing to regenerate OC damage, ranging from the simple repair of articular cartilage to subchondral bone and its smooth interfaces. 3D printing is a layer-by-layer deposition process that allows for precise control of the scaffold’s external shape and internal structure and can effectively mimic the complexity of tissue. The selection of biomaterials, the choice of printing method, and the soundness of the structural design are the main areas to be considered when building OC scaffolds using 3D printing, and all three are interrelated and interdependent. Initially, natural materials were preferred for constructing OC scaffolds, but their poor mechanical strength limited their use in subchondral bone. Subsequently, composites based on natural and synthetic polymers and organic-like materials have been the main focus of research to effectively mimic the mechanical properties of OC bone. EP is the most commonly used fabrication technique. This may be due to the wide availability of EP, the versatility of the materials, and the low cost. The use of combinations of 3D printing techniques is common in the literature and offers a promising approach to better capture different tissue regions, especially in terms of mechanical functionality (Diloksumpan et al., 2020). The structural design of scaffolds has also evolved, with initial research focusing on the field of monophasic scaffolds. However, more and more researchers are focusing on mimicking the layered structure of the subchondral bone, the OC interfaces, and the cartilage, and a series of biphasic, triphasic, and even multiphasic scaffolds have been developed. These scaffolds have also been coupled with tissue-specific cells (osteoblasts, chondrocytes, and stem cells) and appropriate growth factors, to achieve structural gradient mimicry as well as gradient mimicry of the biological environment.

Despite the continuous development of 3D printing technology, research on OC tissue engineering scaffolds for repairing OC defects has gained breakthrough progress, but there are still several problems to be resolved. This includes how to construct gradient scaffolds with tighter interfacial bonding to mimic the gradient structure of natural tissues in terms of structure and function. Although OC-integrated scaffolds are biomimetic in structure and composition, they are not comparable to normal OC tissues at both biological and mechanical levels. And no special materials similar to natural OC tissue have been found. In addition, the repair and regeneration mechanisms of OC-integrated scaffolds have not been thoroughly investigated and cannot yet be elucidated at the microscopic cellular and molecular levels. Animal models are also crucial for clinical translation. Although small animals such as rats and rabbits have the advantage of cost loss, researchers should focus more on large animal models to evaluate the clinical promise of 3D printed scaffolds due to defect size and surgical difficulty. In addition, clinical applications face many regulatory and commercial challenges, and there are currently no clinical trials using 3D printed OC scaffolds to repair articular OC defects. Nevertheless, with the development of emerging materials and 3D printing technology, combined with multidisciplinary and multidisciplinary scientific concepts, OC scaffolds will be further investigated, and perfect regeneration of OC defects will be realized ultimately.
